# Interpreting the Black Box: Interpretable Machine Learning and Systems Pharmacology in Small-Molecule Therapeutics

**DOI:** 10.3390/pharmaceutics18060743

**Published:** 2026-06-16

**Authors:** Huan Zhang, Yangyang Wang, Jihan Wang, Hui Li

**Affiliations:** 1 Department of Joint Surgery, Honghui Hospital, Xi’an Jiaotong University, Xi’an 710054, China; 2 School of Physics and Electronic Information, Yan’an University, Yan’an 716000, China; 3 Department of Immunology, Yan’an Medical College of Yan’an University, Yan’an 716000, China

**Keywords:** interpretable machine learning, systems pharmacology, small-molecule therapeutics, ADME modeling, toxicity prediction, clinical translation

## Abstract

Small-molecule drug development faces high attrition rates driven by complex pharmacokinetics and unforeseen toxicities. While deep learning offers high predictive accuracy, its opaque “black-box” nature hinders mechanistic transparency, clinical trust, and regulatory approval. This review synthesizes how Interpretable Machine Learning, synergized with systems pharmacology, advances this paradigm by enhancing mechanistic transparency in drug development. By providing insights into algorithmic decisions, Interpretable Machine Learning helps researchers identify molecular features that are statistically associated with absorption, distribution, metabolism, and excretion optimization and preemptively mitigate toxicophores, while noting that these associations require experimental validation to establish genuine causality. Furthermore, integrating multi-omics data via Interpretable Machine Learning guides rational polypharmacology, bridging in silico target identification with “dry-wet loop” validations. Crucially, Interpretable Machine Learning accelerates clinical translation by discovering causal biomarkers, refining patient stratification, and generating transparent “Model Cards” to satisfy U.S. Food and Drug Administration/European Medicines Agency regulations. We also discuss future challenges: data heterogeneity, out-of-distribution generalizability, and the evolution toward Causal Artificial Intelligence. Ultimately, the integration of Interpretable Machine Learning provides a framework for more transparent and evidence-based drug design, realizing the promise of precision medicine.

## 1. Introduction

Historically, the development of small-molecule therapeutics relied heavily on empirical high-throughput screening and serendipity—a resource-intensive paradigm plagued by high attrition rates in later clinical stages [1,2]. Over the past decade, however, a profound paradigm shift has occurred, transitioning the field toward algorithm-driven pharmacology. This evolution is largely necessitated by our deepening understanding of disease biology; modern drug targets are rarely isolated entities [3]. Instead, they are deeply embedded within complex, highly interconnected biological networks equipped with robust compensatory feedback loops. As the traditional pursuit of single-target “magic bullets” gives way to multi-target and systems-level strategies, conventional drug development pipelines are increasingly strained by the sheer complexity of the pharmacokinetics (PK) and pharmacodynamics (PD) profiling required for clinical success [4,5].

To navigate this target complexity, the integration of advanced computational methods into therapeutic development has become increasingly critical. State-of-the-art predictive modeling and deep learning (DL) algorithms now offer unprecedented precision in forecasting drug absorption, distribution, metabolism, and excretion (ADME), as well as broader efficacy and safety profiles [6]. By explicitly bridging the gap between molecular-level innovations and macro-level clinical applications, these algorithm-driven strategies directly address critical bottlenecks. They empower researchers to optimize drug metabolism, fine-tune patient-specific dosing, and proactively engineer molecular structures, thereby fulfilling the urgent clinical demand for precision medicine [7,8].

**Figure 1:**
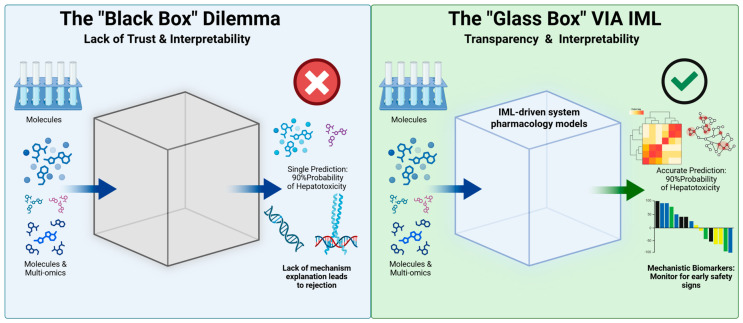
The paradigm shift from the “Black Box” dilemma to the IML-driven “Glass Box” in drug discovery. (**Left**) Traditional deep learning models act as opaque “black boxes.” They ingest molecular structures and multi-omics data to generate accurate but unexplained predictions (e.g., 90% probability of hepatotoxicity). However, the lack of mechanistic explanation leads to clinical distrust and regulatory rejection. (**Right**) Interpretable Machine Learning (IML) transforms this paradigm into a transparent “glass box.” By incorporating visualization tools such as feature importance bar charts and mechanistic attribution lists, IML not only delivers accurate predictions but also provides interpretable insights into the underlying decision-making process. These actionable outputs—including structural optimization suggestions and mechanistic biomarkers—enhance model transparency, support regulatory approval, and enable precision medicine. Created in BioRender. Li, H. (2026) https://BioRender.com/xz17lo6, accessed on 30 May 2026.

Despite these remarkable computational strides, a formidable barrier to clinical translation remains: the “black box” dilemma. While sophisticated DL architectures demonstrate exceptional predictive accuracy, they inherently lack internal transparency [9,10]. In the highly regulated landscape of pharmaceutical development, pure predictive power without mechanistic rationale is fundamentally insufficient. Regulatory bodies (such as the U.S. Food and Drug Administration (FDA) and European Medicines Agency (EMA)), clinicians, and medicinal chemists demand to know why a model flags a specific structural alert for hepatotoxicity, or how a molecule mechanically modulates a multi-target cascade [11,12]. The inability of standard DL models to provide these interpretable biological rationales severely limits their clinical adoption, hampering trust and impeding rational, structure-based optimization. This critical mechanistic gap has catalyzed the emergence of Interpretable Machine Learning (IML). IML represents an important development, shifting the focus from opaque statistical predictions to the generation of biologically actionable insights [13]. By interpreting the algorithmic black box, IML frameworks—when synergistically integrated with systems pharmacology—reposition computational methods from passive screening filters to proactive design architects [14]. As illustrated in Figure 1, by interpreting the algorithmic black box, IML frameworks—when synergistically integrated with systems pharmacology—reposition computational methods from passive screening filters to proactive design architects.

While there have been recent systematic reviews regarding the application of XAI/IML in drug discovery, existing literature predominantly focuses on performance benchmarking of algorithmic architectures [15,16,17]. In contrast to these computational-centric reviews, this work not only addresses the technical landscape but specifically emphasizes the synergistic integration of IML with systems pharmacology to resolve critical scientific challenges within complex biological networks. Furthermore, as global regulatory bodies (e.g., FDA/EMA) increasingly demand greater transparency for AI models, the timeliness of this review lies in its shift from traditional “model performance optimization” toward “clinical translatability and regulatory compliance.” This review aims to provide a conceptual roadmap for pharmaceutical experts, not only identifying how IML facilitates ADME optimization and toxicity prediction, but also exploring how to guide rational polypharmacology through multi-omics data integration. Ultimately, by addressing the “dry-wet loop validation” and “model card” mechanisms, we outline how to translate computation-driven innovation into a foundation of trust for clinical decision-making. By bridging the gap between “black-box predictions” and “biologically interpretable design,” this study provides a unique theoretical framework for the paradigm shift in drug development from experience-driven to evidence-driven precision medicine.

To comprehensively illustrate this synergy, this review systematically navigates the IML-driven drug development pipeline. Section 2 and Section 3 explore how IML explicitly interrogates the physicochemical drivers for ADME optimization and preemptively mitigates structural toxicophores. Section 4 highlights the transition from molecular safety to systemic efficacy, detailing how IML integrates high-dimensional multi-omics data to guide rational polypharmacology and “dry-wet loop” validations. Section 5 bridges the translational gap, demonstrating IML’s indispensable role in discovering causal biomarkers, refining patient stratification, and generating transparent “Model Cards” for regulatory compliance. Finally, Section 6 discusses future frontiers, including overcoming data heterogeneity and the necessary evolution toward Causal AI. Ultimately, this review underscores how IML serves as the vital translational bridge connecting computational molecular design with successful clinical application.

We recognize that the field of IML in drug discovery is still emerging, and most published studies are proof-of-concept or case-based validations. Consequently, this review adopts a narrative synthesis approach, drawing on illustrative examples to highlight methodological trends and translational opportunities. We do not claim statistical superiority of any specific framework, but rather aim to provide a conceptual roadmap for medicinal chemists and computational biologists. Where possible, we explicitly note the lack of head-to-head comparative studies and call for future systematic benchmarking.

## 2. Literature Search Strategy and Selection Criteria

To ensure the comprehensiveness and relevance of this review, a structured literature search was conducted using major scientific databases, including PubMed, Web of Science, and Google Scholar. The search queries primarily focused on the intersection of “Interpretable Machine Learning,” “Systems Pharmacology,” and “Drug Discovery,” utilizing keywords such as “XAI,” “explainable AI,” “model interpretability,” “systems pharmacology,” and “precision medicine.”

We focused our search on literature published between 2019 and 2026 to capture the most recent advancements in this rapidly evolving field. Inclusion criteria prioritized: (1) peer-reviewed research papers and review articles that discuss the methodological development of IML in pharmaceutical research; (2) studies demonstrating the application of XAI/IML in specific drug discovery stages (e.g., ADME prediction, toxicity, and multi-omics integration); and (3) papers discussing the challenges of clinical translation and regulatory compliance.

Exclusion criteria included studies lacking a clear pharmacological application and highly redundant literature that did not provide additional conceptual insights. This selection process ensures that the synthesis presented in this paper reflects both the technical state-of-the-art and the practical requirements for clinical translation.

## 3. Computational Modeling of Pharmacokinetics and ADME

Before delving into specific algorithmic architectures, it is crucial to recognize that optimizing PK and ADME properties remains the cornerstone of successful small-molecule drug development. Accurate early-stage prediction is essential to mitigate costly clinical attrition. This section explores how computational modeling has evolved to address these complex physiological barriers [18].

### 3.1. Advanced Algorithms for Predicting Drug Behavior

Historically, predictive models relied on traditional machine learning algorithms (e.g., Random Forest, Support Vector Machines) using predefined 1D or 2D physicochemical descriptors [19]. While these computationally lightweight models provide robust baselines, they often struggle with “out-of-distribution” generalization when encountering novel chemical scaffolds. The subsequent application of Deep Neural Networks (DNNs) addressed some capacity limitations but remained heavily dependent on the quality of input features and the somewhat rigid grammar of Simplified Molecular Input Line Entry System (SMILES) strings [20,21].

Today, the paradigm is dominated by architectures that naturally learn from molecular topology and sequences. Graph Neural Networks (GNNs) have emerged as the standard for capturing spatial connectivity, treating atoms as nodes and bonds as edges [22]. By dynamically extracting features from 2D graphs, GNNs demonstrate exceptional accuracy in predicting complex physiological barrier penetration, such as blood–brain barrier (BBB) and Caco-2 permeability [23,24]. Concurrently, inspired by the success of natural language processing, Chemical Language Models (e.g., Transformers like ChemBERTa) are revolutionizing the field. By treating SMILES or SELF-Referencing Embedded Strings (SELFIES) as a chemical language, these models leverage self-supervised pre-training on millions of unlabelled molecules [25]. This approach captures long-range molecular dependencies and enables powerful zero-shot or few-shot learning, which is particularly valuable for rare ADME endpoints where high-quality labeled data is scarce [26].

**Figure 2:**
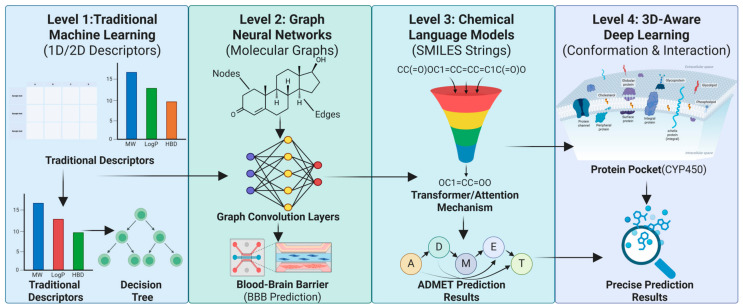
The hierarchical evolution of computational architectures for ADME prediction. The paradigm of predicting pharmacokinetic properties has advanced through four distinct representation levels. (**Level 1**) Traditional machine learning models rely on predefined 1D/2D physicochemical descriptors (e.g., MW, LogP, HBD) to build foundational decision trees. (**Level 2**) Graph Neural Networks (GNNs) capture 2D spatial connectivity by treating atoms as nodes and bonds as edges, significantly enhancing the prediction of physiological barrier penetration, such as the blood–brain barrier (BBB). (**Level 3**) Inspired by natural language processing, Chemical Language Models employ Transformer architectures and attention mechanisms to extract long-range dependencies from SMILES strings for broad Absorption, Distribution, Metabolism, Excretion, and Toxicity (ADMET) profiling. (**Level 4**) 3D-Aware Deep Learning represents the ultimate frontier, integrating spatial conformations and non-covalent interactions within complex enzymatic cavities (e.g., CYP450 protein pockets) to achieve precise dynamic predictions. Created in BioRender. Li, H. (2026) https://BioRender.com/3zm6j0q, accessed on 30 May 2026.

At the cutting edge, the field is rapidly advancing toward 3D-Aware Deep Learning. Many critical pharmacokinetic processes—such as the regioselectivity of Cytochrome P450 (CYP450) metabolism or transporter-mediated efflux (e.g., P-glycoprotein)—are fundamentally driven by 3D spatial conformations and non-covalent interactions [27,28]. Models like SchNet and DimeNet integrate XYZ atomic coordinates and dihedral angles to perfectly capture stereochemistry and distance-dependent interaction mechanisms. While computationally expensive, these 3D architectures represent the ultimate frontier in predicting dynamic in vivo drug behavior, setting the stage for the crucial next step: interpreting these complex algorithms to guide structural optimization [29]. As summarized in Table 1, the computational modeling of ADME properties has undergone a dramatic evolution, transitioning from manual feature engineering to advanced representation learning. Figure 2 conceptually maps this hierarchical progression, illustrating the paradigm shift from traditional 1D descriptors to state-of-the-art 3D-aware architectures.

### 3.2. Interpreting Physicochemical Drivers via Interpretable Machine Learning

Despite the unprecedented predictive accuracy of DL and GNN architectures, their clinical and industrial utility is frequently bottlenecked by their “black-box” nature. For medicinal chemists, simply knowing that a molecule has poor intestinal permeability or rapid metabolic clearance is insufficient; they require actionable insights to modify the structure [30]. This is where IML frameworks can offer value by revealing which molecular features the model has learned to associate with specific ADME outcomes. However, it is crucial to recognize that these IML-derived associations reflect statistical correlations within the training data, not intrinsic biological causality. Nonetheless, when interpreted with caution, these outputs can generate testable hypotheses to guide structural optimization [31,32].

To operationalize these opaque models, IML techniques provide explanations at multiple resolutions. At the global level, methods like SHAP (SHapley Additive exPlanations) and Surrogate Rule Extraction quantify complex trade-offs, such as how lipophilicity (LogP) and topological polar surface area (TPSA) non-linearly influence metabolic stability across a broad chemical series [33,34]. At the local (single-molecule) level, techniques such as Local Interpretable Model-agnostic Explanations (LIME) and Integrated Gradients (IG) pinpoint specific atomic contributors. By superimposing these contribution scores onto molecular graphs, researchers can visually isolate exposed hydrogen bond donors hindering permeability or explicitly identify “metabolic soft spots”—such as oxidizable C-H bonds targeted by CYP450 enzymes [35].

Perhaps the most transformative advancement for medicinal chemistry is the rise of prescriptive IML tools, such as Counterfactual Explanations. Instead of merely highlighting flaws, these algorithms suggest the “minimal structural edits” required to achieve a desired profile (e.g., indicating exactly where a bioisosteric fluorine substitution would shift a molecule’s half-life from short to long) [36]. This transformation shifts the computational paradigm from purely passive screening to proactive, AI-guided structural design, enabling researchers to rationally fine-tune physicochemical profiles and mitigate late-stage attrition risks [37]. It should be noted that the majority of published applications of these IML frameworks are proof-of-concept studies on individual datasets. Direct, head-to-head comparisons of LIME, SHAP, GAT, IG, Counterfactuals, and Surrogate Rules on the same benchmark tasks are largely absent from the literature. Therefore, the “specific applications” listed in Table 2 should be interpreted as representative examples rather than evidence of general superiority. A detailed summary of these diverse IML frameworks and their specific applications in resolving permeability and metabolic liabilities is presented in Table 2.

### 3.3. Limitations and Critical Perspectives on Interpretable Machine Learning

While IML provides essential transparency, it is critical to acknowledge its current limitations to avoid misuse in drug development. As emphasized by Rudin [38], post hoc explanations of black-box models may not always be faithful to the underlying decision-making process, highlighting a fundamental need for inherently interpretable models—rather than mere post hoc interpretations—in high-stakes pharmaceutical decision-making.

Furthermore, users must distinguish between “faithfulness”—how accurately an explanation reflects the model’s internal logic—and “plausibility”—how well an explanation matches human intuition. This distinction is critical because an explanation may seem plausible to a medicinal chemist while being unfaithful to the model’s actual logic, leading to misguided structural optimizations. This challenge is exacerbated by the “disagreement problem,” where different explanation methods, such as LIME and SHAP, can provide conflicting feature importance rankings. Moreover, the known instability of these methods in the presence of highly correlated chemical features can further introduce noise into the design process [39]. Consequently, IML outputs should be regarded primarily as tools for hypothesis generation rather than definitive mechanistic proof, necessitating rigorous iterative validation through the aforementioned “dry-wet loop” [40].

## 4. Computational Tools for Toxicity Prediction and Safety Profiling

While optimizing pharmacokinetic profiles ensures a molecule reaches its intended target, its ultimate clinical viability is dictated by its safety profile. Unanticipated drug toxicity—particularly hepatotoxicity and cardiotoxicity—remains a leading cause of late-stage clinical trial failures and post-market withdrawals [41]. Building upon the predictive modeling concepts discussed in Section 2, this section explores how advanced algorithms, driven by IML and systems pharmacology, are interpreting the black box of computational toxicology.

### 4.1. Transition from Structural Alerts to Algorithm-Driven Safety Profiling

Historically, early-stage toxicity screening relied heavily on heuristic rules, such as the Ashby-Tennant structural alerts, which flag specific chemical fragments associated with mutagenicity or reactive metabolites [42]. While these rule-based systems are easily interpretable, they often lack the necessary contextual awareness of the entire molecular environment [43]. Consequently, they suffer from high false-positive rates, inadvertently halting the development of promising, benign therapeutic candidates [44].

The advent of DL has catalyzed a paradigm shift toward algorithm-driven safety profiling. Moving beyond 1D descriptors and 2D fragments, contemporary DL architectures—such as 3D-Convolutional Neural Networks (CNNs) and transformer-based chemical language models—can now ingest rich, multi-modal data, including 3D spatial conformations and quantum chemical properties [45,46]. As a result, the scope of predictive toxicology has expanded dramatically. By processing vast, high-dimensional datasets, DL models evaluate the holistic structural context rather than isolated fragments [47]. These advanced models implicitly capture complex non-linear interactions, achieving unprecedented predictive accuracy across diverse toxicological endpoints—ranging from localized photoreactivity and skin sensitization to human ether-a-go-go-related gene (hERG) channel inhibition [48].

However, this transition to black-box deep learning resurrects the fundamental dilemma: without understanding why a compound is flagged as toxic, medicinal chemists are left without rational pathways for structural optimization [49]. When a model simply outputs a ‘90% probability of nephrotoxicity’, it provides a diagnostic warning but fails to offer a therapeutic prescription. This opacity represents a critical bottleneck; if researchers cannot identify the specific anionic cluster or lipophilic tail driving the toxicity, they cannot perform the targeted bioisosteric replacements necessary to rescue the compound [50,51].

### 4.2. Precision Toxicophore Identification via Interpretable Machine Learning

To translate algorithmic safety predictions into actionable drug design strategies, IML frameworks are deployed to explicitly identify the structural drivers of toxicity. In the context of safety profiling, IML techniques act as high-resolution diagnostic tools [52]. For example, when a GNN predicts a high risk of Drug-Induced Liver Injury (DILI), attention mechanisms or layer-wise relevance propagation (LRP) can trace the predictive signal back to specific atomic nodes [53,54].

While IML-generated heatmaps can highlight structural features correlated with toxicity predictions, this granular interpretability carries a risk of generating a false sense of mechanistic certainty. In practice, these heatmaps identify statistical associations—a lipophilic tail flagged by a model may indeed be a true toxicophore, or it may simply be a spurious correlate present in the training set. Therefore, IML outputs should guide rather than dictate medicinal chemistry efforts; each algorithmic hypothesis requires experimental confirmation. Instead of abandoning a highly efficacious lead compound due to a generic toxicity flag, chemists can utilize heatmaps generated by IML to pinpoint the exact “toxic liabilities” (e.g., an excessively lipophilic tail or an oxidizable aromatic ring) [55]. Guided by these visual insights, researchers can execute rational bioisosteric replacements, effectively “detoxifying” the molecule while preserving its primary pharmacodynamic properties. Crucially, this IML-driven optimization cycle is no longer limited to classical liabilities like hepatotoxicity or hERG inhibition [56,57]. As the field advances, these transparent frameworks are being systematically applied across a vast spectrum of safety endpoints, ranging from local structural liabilities (e.g., phototoxicity, mutagenicity) to complex multi-target perturbations [58]. Table A1 presents a comprehensive multi-dimensional landscape of these IML applications, detailing the input modalities, extracted physicochemical drivers, and actionable structural mitigation strategies.

### 4.3. Interpreting Off-Target Networks in Systems Toxicology

Toxicity is rarely confined to a single toxicophore; it is frequently the manifestation of unintended multi-target interactions and systemic biological perturbations. To fully capture safety profiles, computational toxicology must evolve into systems toxicology [59,60]. Here, the synergy between IML and systems pharmacology becomes pivotal. Small molecules inevitably interact with a complex network of proteins beyond their primary targets [61]. As highlighted in Table 3, severe liabilities such as hematotoxicity or endocrine disruption frequently arise not from a single reactive moiety, but from promiscuous hinge-binding motifs or pseudo-symmetric scaffolds that inadvertently mimic natural ligands across an entire protein family. Simple structural models cannot predict these off-target effects because they lack the biological context of the human interactome [62].

**Figure 3:**
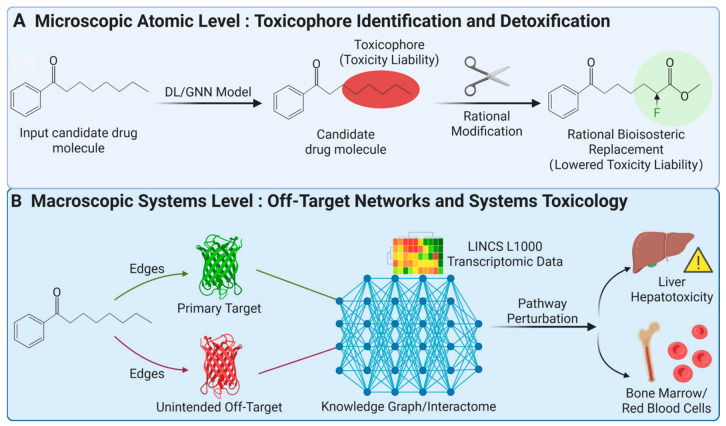
The multi-scale application of IML in predictive toxicology: from atomic resolution to systems-level networks. (**A**) Micro-scale Toxicophore Identification: Deep learning models combined with IML techniques (e.g., attention mechanisms or LRP) generate high-resolution heatmaps. These heatmaps pinpoint specific toxic liabilities (red glowing subgraphs) within a candidate drug molecule, enabling medicinal chemists to execute targeted bioisosteric replacements and rationally detoxify the structure, resulting in a lowered toxicity liability while preserving efficacy. (**B**) Macro-scale Systems Toxicology: To decode complex, multi-target perturbations, IML integrates molecular structures with high-throughput multi-omics data (e.g., transcriptomic signatures) and Knowledge Graphs/Interactomes. This systems-level approach maps unintended off-target interactions through biological cascades, predicting pathway perturbations that manifest as severe organ-specific toxicities (e.g., hepatotoxicity or myelotoxicity). Created in BioRender. Li, H. (2026) https://BioRender.com/bhxq9jh, accessed on 30 May 2026.

By integrating molecular structures with high-throughput biological data—such as large-scale transcriptomic signatures (e.g., LINCS L1000) or interactome databases—AI models can simulate the systemic impact of a drug candidate [63]. IML frameworks, particularly those applied to heterogeneous biological networks (e.g., Knowledge Graphs), can decode these complex perturbations [64]. For instance, by extracting pathway perturbation scores, IML can map a specific solvent-exposed functional group to the unintended inhibition of critical hematopoietic kinases (e.g., JAK2/FLT3), leading to myelotoxicity [65]. Conversely, it can elucidate how binding to an unintended nuclear receptor cascade leads to a teratogenic phenotypic outcome. This system-level interpretation empowers medicinal chemists to exploit subtle sequence differences in binding pockets, designing highly selective substituents that navigate away from toxic nodes while preserving primary efficacy [66,67]. Ultimately, this approach not only predicts organ-specific toxicities with greater biological relevance but also fundamentally bridges the gap between molecular design and complex human pathobiology. This multi-scale computational toxicology framework—spanning from atomic-level toxicophore resolution to systems-level off-target network interpreting—is comprehensively conceptualized in Figure 3.

## 5. Multi-Target and Systems Pharmacology Approaches

While Section 2 and Section 3 demonstrated how advanced computational algorithms can optimize a molecule’s pharmacokinetic journey and anticipate its systemic off-target toxicities, ensuring safety and bioavailability is merely the prerequisite for clinical success. The ultimate challenge lies in achieving sustained therapeutic efficacy [68,69]. For complex, multifactorial diseases—such as oncology, metabolic syndromes, and neurodegenerative disorders—the historical “one drug, one target” paradigm is increasingly faltering against highly robust biological systems equipped with compensatory feedback loops [70]. To overcome this, drug development is fundamentally shifting toward systems pharmacology. By synergizing with IML, researchers are now able to decode the black box of human biology, transitioning from accidental multi-target hits to rational, AI-driven polypharmacology [71].

### 5.1. Interpreting Biological Cascades and Drug-Target Networks

Biological systems operate through highly interconnected and redundant cascades. Traditional computational network pharmacology excels at constructing massive drug-target-disease interaction networks [72]. However, these static representations often devolve into impenetrable topological “hairballs” of nodes and edges, offering limited actionable insights for molecular design [73].

IML can help prioritize which edges in these graphs are most influential in the model’s predictions, offering a roadmap of potential network dynamics. However, such attention-based scores reveal statistical importance within the model’s training distribution, not necessarily true biological signal flow [74]. Experimental perturbation (e.g., gene knockdown followed by phenotypic readout) remains essential to confirm any predicted cascade [75]. From our perspective, the true paradigm shift here is the ability of IML to identify “bottleneck nodes” or “allosteric hubs.” Instead of targeting the most obvious highly connected protein (which often leads to severe systemic toxicity, as discussed in Section 3.3), IML guides researchers to target subtle, vulnerable regulatory hubs within the cascade, achieving maximal phenotypic modulation with minimal collateral network disruption [76].

### 5.2. AI-Driven Multi-Omics Integration for Mechanistic Synergy

The most potent application of systems pharmacology relies on integrating high-dimensional multi-omics data. The fundamental bottleneck in omics analysis is the “curse of dimensionality,” making standard deep learning models highly susceptible to overfitting [77]. IML explicitly resolves this by extracting robust mechanistic synergy. For instance, in identifying clinical biomarkers for Alzheimer’s disease (AD), IML utilizes feature selection wrappers (e.g., Boruta) and SHAP to process millions of transcriptomic data points, successfully isolating pathway enrichments like lipid metabolism dysregulation [78].

Crucially, this synergistic extraction is entirely disease-agnostic. As data availability grows, IML frameworks are scaling across diverse pathological landscapes. Beyond neurodegeneration, researchers are now deploying cross-modal attention mechanisms to integrate host–pathogen genomics for overcoming antimicrobial resistance, or combining kinome profiling with interactome networks to decode compensatory bypass mechanisms in refractory solid tumors [79,80]. By illuminating how specific omics layers interact across these diverse diseases, IML provides the critical biological rationale for combinatorial therapies, explaining why hitting Target A and Target B simultaneously yields superior clinical efficacy.

### 5.3. Rational Polypharmacology and the “Dry-Wet Loop” Validation

Identifying synergistic targets is only half the battle; designing a single small molecule to hit multiple distinct targets simultaneously—known as rational polypharmacology—is a formidable medicinal chemistry challenge. IML bridges this gap by mapping the overlapping chemical space of multiple targets [81]. Techniques like Subgraph Explanations can extract the precise physicochemical features required to maintain affinity for primary targets, while simultaneously penalizing features associated with toxic off-targets. This enables chemists to design highly sophisticated modalities, ranging from dual-kinase hinge binders to bifunctional degraders (PROTACs) and peptidomimetics [82,83].

However, in silico design is merely the blueprint; the ultimate arbiter of efficacy is empirical evidence. The true translational power of this paradigm lies in the iterative “dry-wet loop”. IML-guided polypharmacological designs must be rigorously validated through tailored experimental cascades [84]. This involves transitioning from computational target synergy to in vitro phenotypic assays (such as Patient-Derived Organoids for oncology or multi-electrode arrays for neurotoxicity) and culminating in highly specific in vivo disease models [85]. To underscore the universal applicability of this paradigm, Table 3 presents a comprehensive landscape of IML-driven systems pharmacology that spans 20 distinct pathological contexts and details their multi-omics sources, polypharmacology designs, and essential “ dry-wet loop” validations. The 20 disease contexts summarized in Table 3 are curated from published studies that explicitly applied IML-driven polypharmacology. However, these studies vary widely in data quality, validation rigor, and endpoints. We present them to illustrate the breadth of current exploration, not to rank or compare their relative efficacy.

**Table 3:** Comprehensive Landscape of IML-Driven Systems Pharmacology and Rational Polypharmacology.

Disease Context	High- Dimensional Data Source	IML Synergy Extraction Technique	Identified Synergistic Target Pair	Polypharmacology Design Strategy	Dry-Wet Validation Strategy	References
Alzheimer’s Disease	Transcriptomics + Metabolomics	Feature Selection (Boruta) + SHAP	AChE (Cognition) + MAO-B (Oxidative stress)	Pharmacophore Fusion: Merging indole and benzylpiperidine into a BBB-permeable scaffold.	In vitro enzymatic dual-inhibition; Morris water maze in transgenic mice.	[86]
Solid Tumors (Resistance)	Kinome Profiling+ Interactome	Graph Attention (GAT) on Knowledge Graphs	EGFR (Primary driver) + c-Met (Bypass track)	Hinge-Binding Optimization: Designing dual-kinase inhibitors binding overlapping hinges.	Validation of synergistic apoptosis in Patient-Derived Organoids (PDOs).	[87]
Type 2 Diabetes (T2D)	Lipidomics + Clinical Phenotypes	Subgraph Explanations+ Counterfactuals	GLP-1R (Insulin) + GIPR (Lipid buffering)	Peptidomimetic Dual Agonism: Stabilizing active conformations for both GPCRs.	Intracellular cAMP assays; Oral Glucose Tolerance Tests (OGTT) in DIO mice.	[88]
Antimicrobial Resistance	Bacterial Genomics+ Pathogen Networks	Multi-task Layer-wise Relevance Propagation	PBP (Cell wall) + Efflux Pumps (Clearance)	Zwitterion Design: Dual-action antibiotics evading efflux via zwitterionic charge distribution.	MIC comparative assays in efflux-overexpressing mutant bacterial strains.	[89]
Rheumatoid Arthritis	Proteomics+ Cytokine Networks	Surrogate Rule Extraction (RuleFit)	JAK1 (Inflammation) + SYK (B-cell signaling)	Shape-Based Hybridization: Rigid linkers orienting distinct pharmacophores.	Cytokine release assays in PBMCs; in vivo efficacy in CIA models.	[90]
Heart Failure	Hemodynamics + Transcriptomics	Gradient Boosting Explaners	AT1R (Vasoconstriction) + Neprilysin (Peptide degradation)	Supramolecular Complexation: Co-crystallizing distinct active moieties (ARNI paradigm).	Echocardiography and cardiac remodeling markers in TAC mouse models.	[91]
NASH/MASH	Hepatic Lipidomics + RNA-seq	Pathway Perturbation Scores	FXR (Bile acid regulation) + THR-β (Lipid metabolism)	Dual Allosteric Targeting: Balancing hydrophobicity to enter distinct nuclear receptor pockets.	Liver histology (fibrosis/ steatosis scoring) in diet-induced NASH mice.	[92]
Neuropathic Pain	Ion Channel Interactome	3D-CNN Gradient Weighting	COX-2 (Inflammation) + FAAH	Linker Cleavage Design: Prodrug strategy or overlapping catalytic site targeting.	Von Frey behavioral tests in Chronic Constriction Injury (CCI) models.	[93]
Asthma/COPD	Airway Single-cell RNA-seq	Subgraph Attention Maps	PDE4 (Anti-inflammatory) + M3 Muscarinic (Bronchodilation)	Bifunctional MABA: Integrating an antimuscarinic head with a PDE4-inhibitory tail.	Whole-body plethysmography in ovalbumin-induced guinea pig models.	[94]
Parkinson’s Disease	Striatal Proteostasis Networks	Node Centrality Explanations in GNNs	LRRK2 (Kinase overactivity) + α-Synuclein (Aggregation)	PROTACs/Bifunctional Degraders: Recruiting E3 ligases to specifically degrade LRRK2 complexes.	Rotarod performance and dopaminergic neuron survival in MPTP models.	[95]
Pulmonary Fibrosis (IPF)	Fibroblast Multi-omics	Interpretable Latent Space Arithmetic	TGF-β Receptor (Profibrotic) + PDGFR (Proliferation)	Multi-Kinase Scaffold: Modulating hinge-binding motifs to achieve precise dual-kinase selectivity.	Hydroxyproline quantification and histology in bleomycin-induced lung fibrosis.	[96]
Inflammatory Bowel (IBD)	Gut Microbiome + Host Immunity	Cross-Modal Attention Weights	RIPK1 (Necroptosis) + JAK1 (Cytokine signaling)	Gut-Restricted Design: Elevating TPSA and MW, minimizing systemic absorption.	Colon length measurement and endoscopic scoring in DSS-induced colitis.	[97]
Acute Leukemia (AML)	Bone Marrow Microenvironment	SHAP Dependence Plots	FLT3-ITD (Proliferation) + Bcl-2 (Anti-apoptosis)	Orthogonal Targeting: Combining kinase inhibition with BH3-mimetic protein- protein disruption.	Ex vivo blast viability assays; Survival analysis in PDX models.	[98]
Broad- Spectrum Viral	Viral replication interactomes	Motif-based GNN Explaners	RdRp (Viral replication) + 3CLpro (Viral cleavage)	Covalent Warhead Tuning: Reversible electrophiles targeting both active sites.	Viral titer reduction (plaque assays) in primary human airway epithelial cells.	[99]
Major Depression (MDD)	Neuroimaging + Synaptic Networks	Integrated Gradients (IG)	SERT (Serotonin reuptake) + 5-HT1A (Autoreceptor)	SPARI Paradigm: Blending an SSRI pharmacophore with an indole-based partial agonist.	Forced Swim Test (FST) and microdialysis of extracellular serotonin in vivo.	[100]
Osteoarthritis (OA)	Osteoarthritis (OA)	RuleFit Algorithm	MMP-13 (Cartilage degradation) + COX-2 (Pain/Inflammation)	Intra-Articular Retention: Modifying lipophilicity to prolong synovial half-life of the dual inhibitor.	Joint histopathology (OARSI scoring) in Destabilization of Medial Meniscus (DMM) models.	[101]
Chronic Kidney Disease	Renal single- nucleus RNA-seq	Layer-wise Relevance Propagation	SGLT2 (Glucose/ Sodium symport) + AT1R (Hemodynamics)	Pharmacokinetic Matching: Designing a single molecule or fixed-dose combo with aligned renal clearance.	Albuminuria quantification and GFR monitoring in diabetic db/db mice.	[102]
Psoriasis/ Dermatitis	Skin Biopsy Spatial Transcriptomics	Feature Attribution Methods	TYK2 (IL-23 signaling) + PDE4 (Intracellular cAMP)	Topical Penetration Optimization: Fine-tuning molecular weight and LogP for stratum corneum permeation.	Epidermal thickness reduction in Imiquimod- induced skin inflammation models.	[103]
Glaucoma	Trabecular Meshwork Proteomics	GAT Attention Overlays	ROCK (Cytoskeleton relaxation) + FP Receptor (Uveoscleral outflow)	Nitric Oxide Donating Moieties: Attaching an NO-donating group to a receptor agonist scaffold.	Sustained Intraocular Pressure (IOP) reduction monitoring in non-human primates.	[104]
Cystic Fibrosis	CFTR Proteostasis Networks	Causal Inference Networks	CFTR Corrector (Folding) + CFTR Potentiator (Channel opening)	Allosteric Modulator Synergy: Targeting distinct, non- overlapping domains on the mutant CFTR protein.	Ussing chamber assays evaluating chloride transport in patient-derived nasal epithelia.	[105]

## 6. Clinical Translation and Precision Medicine

The preceding sections demonstrated how IML interrogates the “black box” of preclinical drug discovery. However, the ultimate crucible for any therapeutic is the human clinical trial. Even the most exquisitely designed multi-target molecule will fail if administered to an unstratified patient population. In this critical phase, IML transitions from a structural design tool to a clinical navigator [106,107]. From our perspective, the true value of IML in this stage is not merely computational throughput, but its ability to restore biological causality to a field increasingly dominated by opaque statistical correlations.

### 6.1. Interpreting Mechanistic Biomarkers from Systems-Level Data

The leap from controlled laboratory models to highly heterogeneous human clinical cohorts introduces unprecedented data noise. Standard deep learning models applied to clinical omics often identify hundreds of statistically significant but biologically irrelevant “bystander” genes [108,109]. A prevalent critique in contemporary computational biology is that these high-dimensional models frequently devolve into “fishing expeditions”—yielding predictive signatures that lack mechanistic rationale [110].

IML frameworks directly address this translational bottleneck by extracting a sparse set of causal biomarkers. By utilizing feature attribution methods (such as SHAP) on clinical trial data, researchers can isolate the true pathological drivers from background physiological noise [111]. We argue that IML shifts the paradigm from finding “what” correlates with a disease to understanding “why” it occurs. Furthermore, IML bridges the gap between discovery and routine clinical utility [112]. By employing discretization algorithms, IML can convert complex risk scores into actionable clinical thresholds, laying the essential groundwork for Companion Diagnostics (CDx). This ensures that biomarker discovery is driven by clinical applicability rather than pure computational curiosity [113].

### 6.2. IML-Guided Patient Stratification and Precision Therapeutics

The mechanistic biomarkers discovered in the previous step serve as the foundation for IML-guided patient stratification. The rational polypharmacological agents designed in Section 4 exhibit their superior efficacy only in patient subpopulations harboring those specific pathway dysregulations [114]. IML algorithms act as high-precision matching engines between patient profiles and drug mechanisms [115].

However, a critical commentary must be made regarding the “Precision Paradox.” While extreme patient stratification maximizes therapeutic efficacy, it risks shrinking the target population to commercially unviable micro-cohorts. We assert that the role of IML is to find the optimal “sweet spot”—balancing precision efficacy with sufficient cohort size [116]. Beyond static stratification, IML empowers dynamic clinical development through Adaptive Trial Designs. By leveraging counterfactual survival analysis mid-trial, IML provides transparent, evidence-based rationales for dropping futile treatment arms or expanding successful ones [117]. Furthermore, integrating pharmacogenomic profiles optimizes individualized dosing strategies, maximizing success rates while minimizing systemic toxicities.

### 6.3. Overcoming the “Black Box” Barrier in Clinical Regulatory Approval

Despite the profound computational advances, the most formidable barrier to clinical translation is often regulatory skepticism. Agencies like the FDA and EMA operate on the principle of biological plausibility and risk mitigation [118]. A “black box” AI model is fundamentally incompatible with stringent clinical safety standards and physician accountability [119].

**Figure 4:**
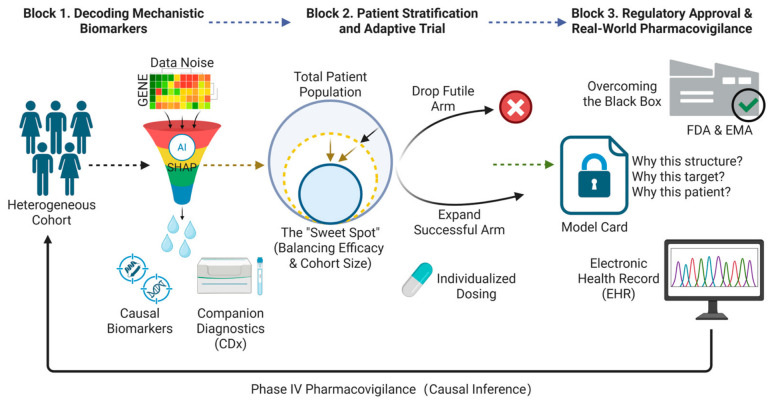
The IML-driven clinical translation framework: from causal biomarker discovery to regulatory approval and pharmacovigilance. The integration of Interpretable Machine Learning (IML) navigates the critical phases of clinical development. (**Left**) IML extracts causal mechanistic biomarkers from noisy, heterogeneous clinical cohorts, transforming opaque statistical correlations into actionable Companion Diagnostics (CDx). (**Middle**) During clinical trials, IML guides patient stratification to navigate the “Precision Paradox”—finding the optimal sweet spot between high efficacy and a viable cohort size. Concurrently, it empowers adaptive trial designs by identifying futile or successful treatment arms mid-trial for individualized dosing. (**Right**) To overcome regulatory skepticism and open the “black box,” IML provides transparent Model Cards that elucidate biological rationales. This interpretability extends seamlessly into Phase IV, where causal inference on real-world Electronic Health Records continuously refines post-market pharmacovigilance. Created in BioRender. Li, H. (2026) https://BioRender.com/31s47ot, accessed on 30 May 2026.

When a sponsor submits an AI-discovered molecule, IML provides the essential “model cards” and mechanistic narratives required by emerging regulatory guidelines. It answers the critical “Why?”—why this structure, why this target, and why this patient [120]. It is our perspective that interpretability should no longer be treated as a “post hoc patch” applied to a black-box model just to satisfy regulators. Instead, IML must be integrated as a foundational design principle from day one (ante-hoc interpretability) [121]. Importantly, this interpretability extends beyond initial approval into Phase IV Pharmacovigilance. By utilizing causal inference networks on real-world electronic health records, IML can disentangle true drug-induced adverse events from underlying disease progression, enabling the proactive and precise updating of safety warnings [122]. To holistically illustrate this paradigm, the comprehensive framework detailing how IML addresses the entire clinical lifecycle—from biomarker interpreting to regulatory compliance and post-market pharmacovigilance—is visually conceptualized in Figure 4.

## 7. Challenges and Future Directions

### 7.1. A Paradigm Shift in Small-Molecule Therapeutics

The integration of advanced computational algorithms into the pharmaceutical pipeline represents a fundamental paradigm shift rather than a mere technological upgrade. As explored throughout this review, the historical reliance on “black-box” models or heuristic rules frequently culminated in costly late-stage attrition. By interpreting the black box, IML has repositioned computational methods from passive screening filters to proactive, rational design architects. From precisely fine-tuning physicochemical properties and preemptively mitigating systemic toxicities, to orchestrating rational polypharmacology across biological networks and establishing regulatory trust through transparent clinical stratification, IML serves as the critical connective tissue. It transforms opaque predictive probabilities into actionable medicinal chemistry insights and robust clinical “Model Cards,” thereby fundamentally accelerating the efficiency and success rate of small-molecule drug development.

### 7.2. Data Heterogeneity and the Generalizability Bottleneck

Despite these profound advancements, realizing the full translational potential of IML is currently constrained by formidable challenges. The foremost obstacle is the availability and quality of data. Deep learning and IML frameworks are inherently data-hungry; however, pharmaceutical data remains heavily siloed, fragmented, and plagued by experimental noise. Public databases often lack negative data (failed experiments), which is crucial for training unbiased models. Furthermore, models trained on highly specific, historically explored chemical spaces frequently struggle with “Out-of-Distribution” (OOD) generalizability. When tasked with evaluating entirely novel chemical scaffolds or previously uncharacterized allosteric pockets, the predictive accuracy of these models drastically declines. Future efforts must prioritize the development of Federated Learning architectures—enabling multi-institutional model training without compromising proprietary data—and the creation of standardized, high-quality, multi-omics open datasets.

### 7.3. Toward Causal Inference and Autonomous Drug Discovery

Looking toward the horizon, the next evolution of computational pharmacology must transcend the limitations of current interpretability. Present IML techniques, such as SHAP or LIME, are predominantly post hoc; they excel at explaining what features a model relied upon based on statistical correlations, but they do not inherently understand the underlying biological physics. The field must pivot from correlation analysis to true Causal Inference. Future “Causal AI” frameworks will explicitly model the cause-and-effect relationships within biological networks, predicting not just that a molecule will inhibit a kinase, but simulating the exact downstream phenotypic consequences of that perturbation. Ultimately, integrating these causal models with Large Language Models (LLMs) and automated robotic synthesis will pave the way for “Autonomous AI Scientists”—closed-loop systems capable of designing a molecule, synthesizing it, testing it in vitro, and autonomously updating their causal understanding in real-time, thereby fully realizing the promise of systems pharmacology.

## 8. Conclusions

The integration of IML with systems pharmacology represents a significant development in small-molecule therapeutics, shifting the computational paradigm from opaque predictive screening toward more transparent methods for drug design. By interpreting the black box, IML assists researchers in mapping physicochemical drivers for structural optimization, mitigating toxicophores, and exploring polypharmacology within complex biological networks. Furthermore, extending this interpretability into the clinical domain may provide mechanistic insights and regulatory ‘model cards’ that support patient stratification and clinical approval. However, IML is not a panacea; its current utility remains limited by data heterogeneity, out-of-distribution generalizability, and the challenge of moving beyond statistical correlation toward causal inference. Rather than replacing the expertise of medicinal chemists and clinicians, IML functions as a translational tool—potentially fostering trust and addressing late-stage attrition risks by providing a more evidence-based framework for therapeutic discovery. Finally, the conclusions drawn in this review are based on a qualitative synthesis of available literature. Given the current lack of systematic meta-analyses and standardized benchmarking, our assessment of different frameworks serves as directional guidance rather than definitive evidence. Future work should prioritize reproducible, head-to-head comparisons to strengthen the evidence base for IML-driven drug design.

## Figures and Tables

**Table 1:** Evolution and Comparison of Computational Approaches for ADME Prediction.

Algorithm Type	Input Representation	Key Advantages	Primary Limitations	Typical ADME Applications
Traditional Machine Learning (ML)& Ensembles (Random Forest, XGBoost, SVM)	1D/2D Physicochemical Descriptors, Molecular fingerprints (e.g., Morgan)	Computationally lightweight; highly robust for tabular data; inherently less prone to overfitting.	Cannot extract novel structural features directly; poor generalization to novel chemical scaffolds (Out-of-Distribution).	Baseline rapid screening for basic properties (LogP, aqueous solubility, plasma protein binding).
Deep Neural Networks (DNNs, 1D-CNNs)	SMILES strings, numerical descriptor vectors	High capacity for massive datasets; capable of modeling complex non-linear pharmacological relationships.	Prone to the “black-box” dilemma; relies heavily on the quality of input feature engineering.	High-throughput multi-task ADMET screening across large corporate chemical libraries.
Chemical Language Models (Transformers, e.g., ChemBERTa)	SMILES or SELFIES sequences	Leverages self-supervised pre-training on millions of unlabelled molecules; captures long-range sequential dependencies.	Struggles with stereochemistry and 3D realities; requires immense computational power for pre-training.	Zero-shot or few-shot learning for rare ADME endpoints; broad chemical space exploration.
2D Graph Neural Networks (GCNs, GATs, MPNNs)	2D Molecular Graphs (Nodes = Atoms, Edges = Bonds)	Naturally preserves local topological structure; dynamic feature extraction from atomic connectivity.	Fails to distinguish conformational isomers and non-covalent spatial interactions.	Predicting complex barrier permeability (e.g., BBB, Caco-2); identifying metabolic soft spots.
3D-Aware Deep Learning (3D-GNNs, e.g., SchNet, DimeNet)	3D Conformations (XYZ atomic coordinates, bond angles, torsions)	Perfectly captures spatial arrangements, stereochemistry, and distance-dependent interaction mechanisms.	Extremely high computational cost for 3D conformer generation; sensitive to the initial conformation input.	Regioselectivity of CYP450 metabolism; complex transporter-mediated efflux prediction (e.g., P-gp).

**Table 2:** Comprehensive Comparison of IML Frameworks for Resolving Permeability and Metabolic Liabilities.

IML Framework	Resolution & Output	Key Advantages	Primary Limitations	Specific Application (Permeability & Stability)
LIME	Local: Substructure perturbation weights.	Model-agnostic; computationally light; easy to implement for any black-box model.	Explanations can be unstable; ignores complex correlations between global features.	Permeability: Identifying specific exposed polar groups (e.g., H-bond donors) that acutely hinder Caco-2 permeability in a single lead compound.
SHAP	Global & Local: Unified feature importance plots.	Solid mathematical foundation (Game Theory); ensures consistent and fair feature attribution.	Computationally expensive for large datasets; assumes feature independence (often violating chemical reality).	Metabolic Stability: Quantifying the non-linear trade-off between lipophilicity (LogP) and intrinsic clearance across an entire chemical series.
Graph Attention (GATs)	Local: Heatmaps on 2D/3D molecular graphs.	Native to molecular topology; directly maps to a chemist’s structural intuition.	Attention weights do not always strictly align with true predictive feature importance.	Metabolic Stability: Visually pinpointing “Metabolic soft spots” (e.g., specific oxidizable C-H bonds targeted by CYP450) to guide bioisosteric replacement.
Integrated Gradients (IG)	Local: Atomic-level contribution scores.	Satisfies the “completeness” axiom; highly detailed gradient tracking from output to input.	Requires a differentiable, “ white-box” model architecture; noisy gradients can mislead.	Permeability: Highlighting precise topological polar surface area contributors preventing blood–brain barrier crossing.
Counterfactual Explanations	Prescriptive: “What-if” structural edits.	Highly actionable; directly outputs specific modifications needed to change a prediction.	May suggest synthetically infeasible molecules if not strictly constrained by chemical rules.	Stability & Permeability: Suggesting minimal edits (e.g., “Adding a fluorine atom at C4 shifts half-life from short to long”).
Surrogate Rules (e.g., RuleFit)	Global: Human-readable Boolean rules.	Extreme transparency; generates actionable “Lipinski-like” design guidelines.	Sacrifices some predictive accuracy; struggles to capture highly non-linear 3D spatial dynamics.	Multidimensional Optimization: Extracting novel, data-driven rules to simultaneously balance permeability and metabolic stability.

## Data Availability

No new data were created or analyzed in this study. Data sharing is not applicable to this article.

## Abbreviations

The following abbreviations are used in this manuscript:

ADME	Absorption, Distribution, Metabolism, and Excretion
ADMET	Absorption, Distribution, Metabolism, Excretion, and Toxicity
AI	Artificial Intelligence
BBB	Blood–Brain Barrier
CDx	Companion Diagnostics
CNN	Convolutional Neural Network
DILI	Drug-Induced Liver Injury
DL	Deep Learning
DNN	Deep Neural Network
EMA	European Medicines Agency
FDA	U.S. Food and Drug Administration
GAT	Graph Attention Network
GNN	Graph Neural Network
hERG	Human ether-a-go-go-related gene
IG	Integrated Gradients
IML	Interpretable Machine Learning
KG	Knowledge Graph
LIME	Local Interpretable Model-agnostic Explanations
LRP	Layer-wise Relevance Propagation
OOD	Out-of-Distribution
PD	Pharmacodynamics
PK	Pharmacokinetics
PROTACs	Proteolysis Targeting Chimeras
SELFIES	SELF-Referencing Embedded Strings
SHAP	SHapley Additive exPlanations
SMILES	Simplified Molecular Input Line Entry System
TPSA	Topological Polar Surface Area

## Appendix A

**Table A1:** Multi-Dimensional Landscape of IML-Guided Toxicophore Identification and Structural Mitigation.

Toxicity Endpoint	Mechanism	Key Input Modality	Dominant IML Technique	Extracted Structural/ Physicochemical Drivers	IML-Guided Mitigation Strategy (Action)
Hepatotoxicity (DILI)	Mitochondrial toxicity; Reactive metabolite formation	2D/3D Molecular Graphs	Graph Attention (GAT), SHAP	High lipophilicity (LogP > 3); Specific oxidizable rings (e.g., anilines, thiophenes).	Action: Directed halogenation to block metabolic soft spots; lowering LogP via polar group insertion.
Cardiotoxicity (hERG)	K + channel blockade	3D Conformations, Pharmacophores	Layer-wise Relevance Propagation (LRP), IG	High basicity (pKa > 8) interacting with specific hydrophobic distances.	Action: Attenuating basicity via amine-to-amide conversion, lowering lipophilicity, disrupting planar geometry, inducing steric clashes within the hERG pore.
Mutagenicity/Genotoxicity	DNA intercalation; Covalent binding	SMILES, 2D Graphs	Subgraph Explanations, Counterfactuals	Hidden Michael acceptors, epoxides, or aromatic nitro groups missed by traditional rules.	Action: Prescriptive scaffold hopping; saturation of reactive double bonds.
Nephrotoxicity	Accumulation via renal transporters (e.g., OAT1/3, OCT2)	Molecular Descriptors, 2D Graphs	LIME, Surrogate Rule Extraction	Specific anionic/cationic clusters paired with high molecular weight.	Action: Altering ionization state at physiological pH (pKa tuning); reducing overall molecular footprint.
Neurotoxicity/CNS Toxicity	Unintended CNS target binding (e.g., GABA_A antagonism)	BBB Permeability + Target Structure	Multi-task GNN Attention	Excessive lipophilicity driving BBB crossing + specific rigid planar scaffolds.	Action: For peripheral targets, intentionally increasing TPSA to prevent BBB crossing; enriching sp3 hybridization (Fsp3).
Phospholipidosis	Lysosomal trapping	1D/2D Physicochemical descriptors	SHAP, Decision Trees	Cationic Amphiphilic Drug (CAD) traits: High ClogP paired with a highly basic center.	Action: Breaking the amphiphilic nature: reducing lipophilicity or neutralizing the basic nitrogen (pKa < 7).
Endocrine Disruption/ Teratogenicity	Nuclear receptor agonism/ antagonism	3D Protein-Ligand Complexes	3D-CNN Gradient weighting	Phenolic rings mimicking natural hormones (e.g., estradiol); specific hydrophobic tail lengths.	Action: Disrupting pseudo-symmetry; introducing steric bulk to prevent proper receptor pocket folding.
Phototoxicity	UV-induced ROS generation; Photoreactivity	3D Conformations, Quantum Chemical Descriptors	Gradient Boosting Explaners, GAT	Pathway Perturbation Scores, Multi-task Attention	Action: Disrupting extended conjugation (e.g., breaking planarity); adding electron-withdrawing groups to widen the gap.
Skin Sensitization	Covalent binding to skin proteins	Reactivity descriptors, 2D Molecular Graphs	Surrogate Rule Extraction, Subgraph Explanations	Specific electrophilic functional groups (e.g., aldehydes, unhindered Michael acceptors).	Action: Increasing steric hindrance around the electrophilic center; replacing with softer or reversible electrophiles.
Hematotoxicity/Myelotoxicity	Off-target kinase inhibition; Progenitor cell apoptosis	Transcriptomic signatures, Protein-Ligand Graphs	Pathway Perturbation Scores, Multi-task Attention	Promiscuous hinge-binding motifs cross-reacting with critical hematopoietic kinases.	Action: Optimizing solvent-exposed regions to exploit specific kinase pocket geometries, thereby enhancing selectivity.
Systemic Off-Target Effects	Broad network perturbation	Multi-omics, Knowledge Graphs	Pathway Perturbation Scores	Promiscuous hinge-binding motifs; broad polypharmacological connectivity.	Action: Exploiting subtle sequence differences in kinase binding pockets to design highly selective substituents.
